# Enhanced proton acceleration from an ultrathin target irradiated by laser pulses with plateau ASE

**DOI:** 10.1038/s41598-018-20948-3

**Published:** 2018-02-07

**Authors:** Dahui Wang, Yinren Shou, Pengjie Wang, Jianbo Liu, Chengcai Li, Zheng Gong, Ronghao Hu, Wenjun Ma, Xueqing Yan

**Affiliations:** 1 State Key Laboratory of Nuclear Physics and Technology, Peking University, Beijing, 100871 China; 2 State Key Laboratory of Laser Interaction with Matter, Northwest Institute of Nuclear Technology, Xi’an, 710024 China; 3 Collaborative Innovation Center of Extreme Optics, Shanxi University, Taiyuan, Shanxi 030006 China

## Abstract

We report a simulation study on proton acceleration driven by ultraintense laser pulses with normal contrast (10^7^–10^9^) containing nanosecond plateau amplified spontaneous emission (ASE). It’s found in hydrodynamic simulations that if the thickness of the targets lies in the range of hundreds nanometer matching the intensity and duration of ASE, the ablation pressure would push the whole target in the forward direction with speed exceeding the expansion velocity of plasma, resulting in a plasma density profile with a long extension at the target front and a sharp gradient at the target rear. When the main pulse irradiates the plasma, self-focusing happens at the target front, producing highly energetic electrons through direct laser acceleration(DLA) building the sheath field. The sharp plasma gradient at target rear ensures a strong sheath field. 2D particle-in-cell(PIC) simulations reveal that the proton energy can be enhanced by a factor of 2 compared to the case of using micrometer-thick targets.

## Introduction

Proton acceleration by the interaction of ultraintense, ultrashort laser pulses with matter has obtained high energy and short duration proton beams^1,2^. For applications in the fields of medical therapy, inertial confinement fusion (ICF), material irradiation effects and proton imaging^3,4^, several acceleration schemes including target normal sheath acceleration (TNSA)^5–8^, radiation pressure acceleration (RPA)^9–11^, and break-out after burner (BOA)^12,13^ were widely studied. In these schemes, the presence of nanosecond ASE pedestal leaked from the regenerative amplifier can significantly influence the acceleration process. Many researches have studied the effects on different mechanisms of ion acceleration by ASE theoretically and experimentally^14–19^. For a typical Ti:sapphire laser, the ASE has a contrast of 10^7^–10^9^ and duration of a few nanosecond. Under the interaction of ASE, a preplasma will be created at the target front^20–24^. Meanwhile, the shock launched by the ASE, after propagating through the target, will give rise to an expanding plasma at the rear side of the target^17,25–27^. It is found that the preplasma normally contributes positively to the acceleration as it enhances the laser absorption^16,28–32^, but the plasma at the rear side of the target, even with *μm* scale length, can significantly reduce the sheath field^33–37^. As a result, the presence of ASE in most cases is harmful to proton acceleration. Several techniques such as plasma mirrors^38,39^, optical parametric chirped pulse amplification (OPCPA) technique^40^ and plasma shutters^41^ have been employed to suppress ASE. But most of them take the price of energy loss for the laser pulses.

In this letter,we report on the proton acceleration by irradiating targets using normal contrast (10^7^–10^9^) laser pulses^15^ containing nanosecond plateau amplified spontaneous emission (ASE). We found in hydrodynamic simulations^42^ that if the thickness of target was in the range of hundreds nanometer, the plasma gradient at the target rear was unexpectedly short compared to micrometer-thick targets. Relativistic PIC simulations^43^ revealed that the maximum proton energy obtained from these ultrathin targets was 2 times as much as that of micrometer-thick target. This result can be explained that for ultrathin targets, the displacement resulting from ablation pressure of ASE exceeds the forward expansion of the plasma at the target rear. As a result, the plasma density profile becomes asymmetry, and a density spike, still overdense meanwhile, moves forwardly as a whole. In this case, the plasma gradient at the rear surface of the moving target is very short, which is highly favorable to proton acceleration. Systematic study reveals that the optimal target thickness depends on the ASE intensity. At the optimal thickness, the dependence of the maximum proton energy on the main pulse intensity was also investigated.

## Results

**Figure 1:**
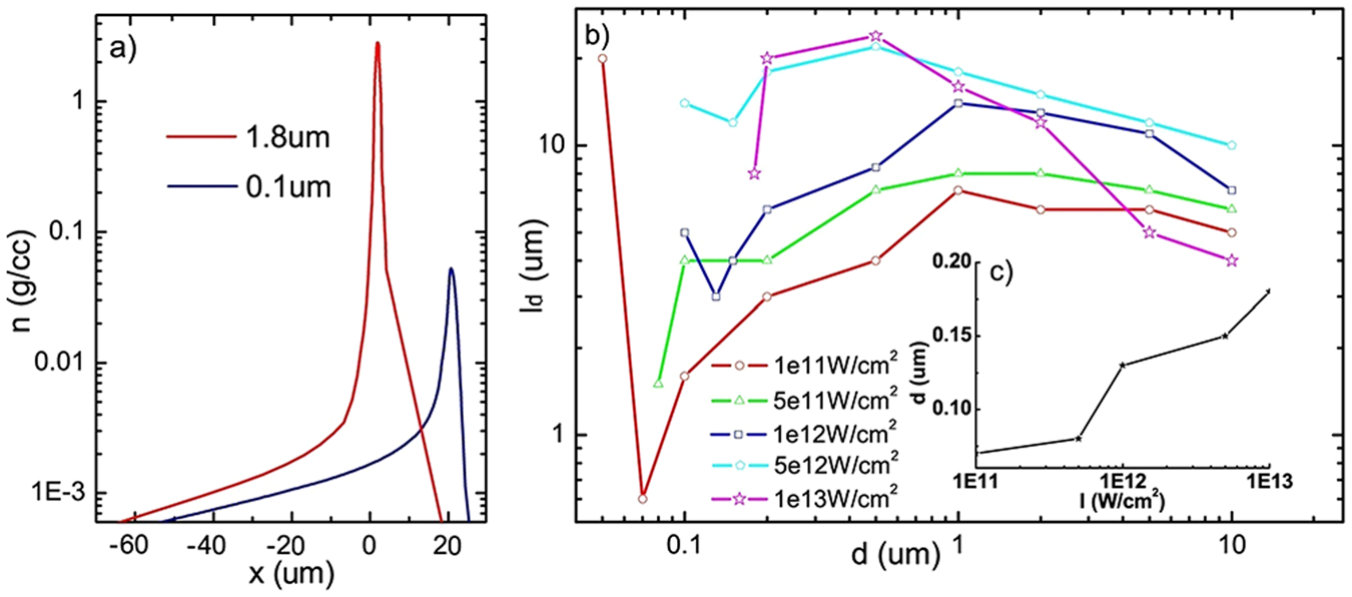
(**a**) The plasma density profiles from MULTI simulations for different thickness of targets irradiated by ASE at intensity of 10^12^ *W*/*cm*^2^. The red line and blue line are the results of 1.8 *μm* and 0.1 *μm* respectively.The laser originates from the left side. (**b**) Plasma gradient scale length at the target rear versus the thickness of the target irradiated by different ASE intensity. (**c**) The dependence of optimal target thickness with minimum gradient scale length on ASE intensity.

Hydrodynamic simulations were performed to study the evolution of the targets under irradiation of ASE. The ASE was set such that the pulse duration is 0.5 *ns* and the intensity ranges from 10^11^ *W*/*cm*^2^ to 10^13^ *W*/*cm*^2^. Figure 1(a) shows the plasma density distribution along the laser direction at intensity of 10^12^ *W*/*cm*^2^ for aluminum target with thickness of 0.1 *μm* and 1.8 *μm*. The density profile at the front of the targets can be expressed by *n*_*e*_ = *n*_*c*_*exp*$(\frac{x-{x}_{c}}{{l}_{f}})$ for *x* < *x*_*c*_, where *n*_*c*_ is the critical density, *x*_*c*_ the position of critical-density layer,and *l*_*f*_ the scale length of the plasma at the target front. The density distribution at the target rear can be described by the formula *n*_*e*_ = *n*_0_*exp*$(-\frac{{l}_{r}}{{l}_{d}})$^44^, where *l*_*r*_ is the distance away from the unperturbed region, *l*_*d*_ is plasma scale length at the target rear. *l*_*f*_ and *l*_*d*_ are both crucial to the proton acceleration. It was reported that the optimal thickness of the targets, at which the combination of *l*_*f*_ and *l*_*d*_ was optimal for proton acceleration, was 3.6 *μm*/*ns* × *τ*_*ASE*_ in previous study^16^. Therefore, we chose a 1.8 *μm* thick target matching our 0.5 *ns* ASE as a comparison to 0.1 *μm* targets. By fitting obtained density profiles in the simulations, it’s found that *l*_*f*_/*l*_*d*_ of 0.1 *μm* and 1.8 *μm* targets is 62/2.5 *μm* and 52/15 *μm* respectively. Although *l*_*f*_ of the two cases are similar, the difference of *l*_*d*_ is drastic. To systematically investigate the influence of ASE on *l*_*d*_, we varied the ASE intensity *I*_*ASE*_ and performed serials of simulations. *l*_*d*_ is shown as a function of *d* for different *I*_*ASE*_ in Fig. 1(b), where *d* is the thickness of the target. It’s found that for a given *I*_*ASE*_, there was an optimal thickness in the range of a few hundreds nanometer, where *l*_*d*_ is minimal. This optimal thickness linearly scales up with the increment of ASE intensity as shown in Fig. 1(c). The value of *l*_*d*_ at the optimal thickness scales up the intensity of ASE with a minimal value of 0.6 *μm* for 0.07 *μm* targets.

**Figure 2:**
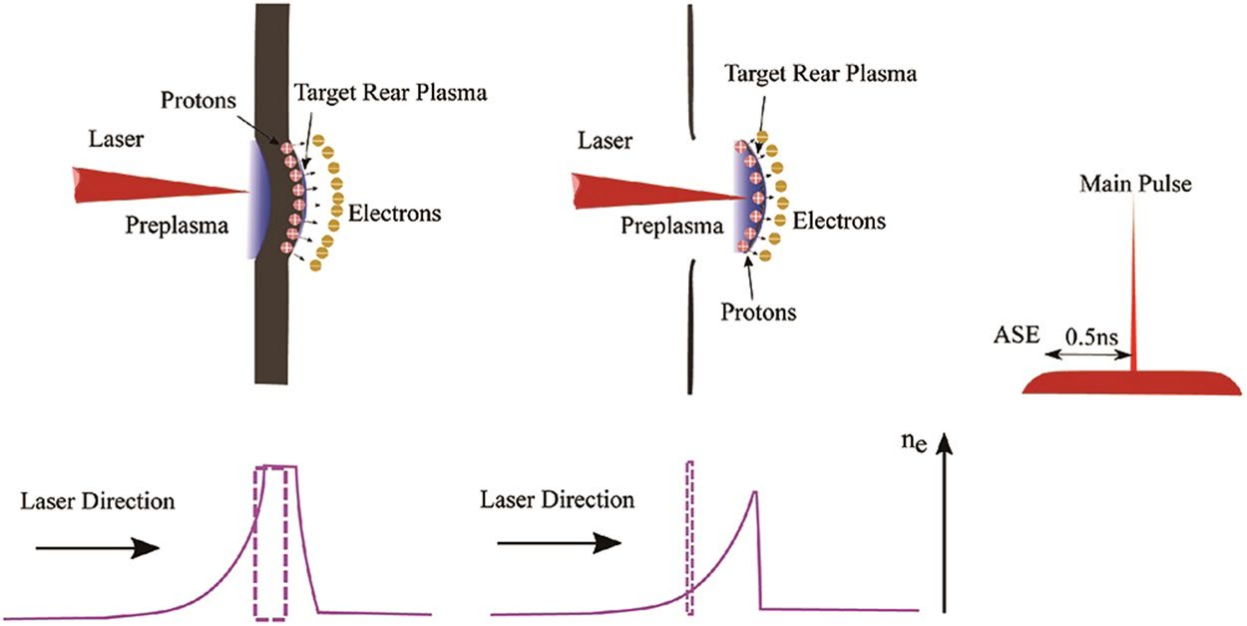
Schematic of proton acceleration by 0.1 *μm* and 1.8 *μm* target interaction with laser pulse containing ASE.

Figure 2 schematically illustrates the acceleration process for the cases of ultrathin and micrometer-thickness foils irradiated by the laser pulses with plateau ASE. For a micrometer-thick target, preplasma with scale length of tens of micrometer is formed at the target front, in together with deformation and a plasma with sufficient extension at the target rear. The whole target has a small displacement. In the case of hundreds-nanometer-thin target, preplasma with similar scale length is formed at the target front. However, the whole target move a long distance along the laser direction with a smaller scale length plasma at the target rear. After the ASE, the main pulses interact with the expanded plasma at the target front and generate hot electrons. When these electrons leave the targets, a sheath field is established at the rear side of the targets. The scale length of plasma at both sides of the targets will impose significant influence on the strength of the sheath field.

**Figure 3:**
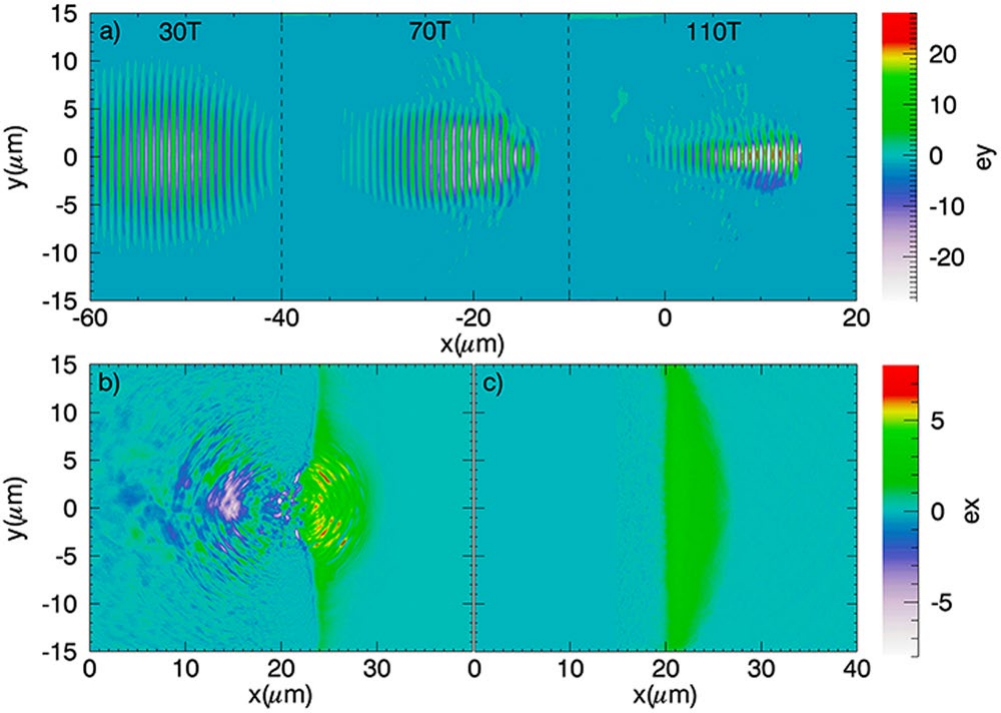
(**a**) The normalized laser electric field Ey at 30 *T*, 70 *T* and 110 *T*. The maximum normalized electric field Ex of 0.1 *μm* (**b**) and 1.8 *μm* (**c**) targets respectively. The origin targets before ASE locate at the position of x = 0.

**Figure 4:**
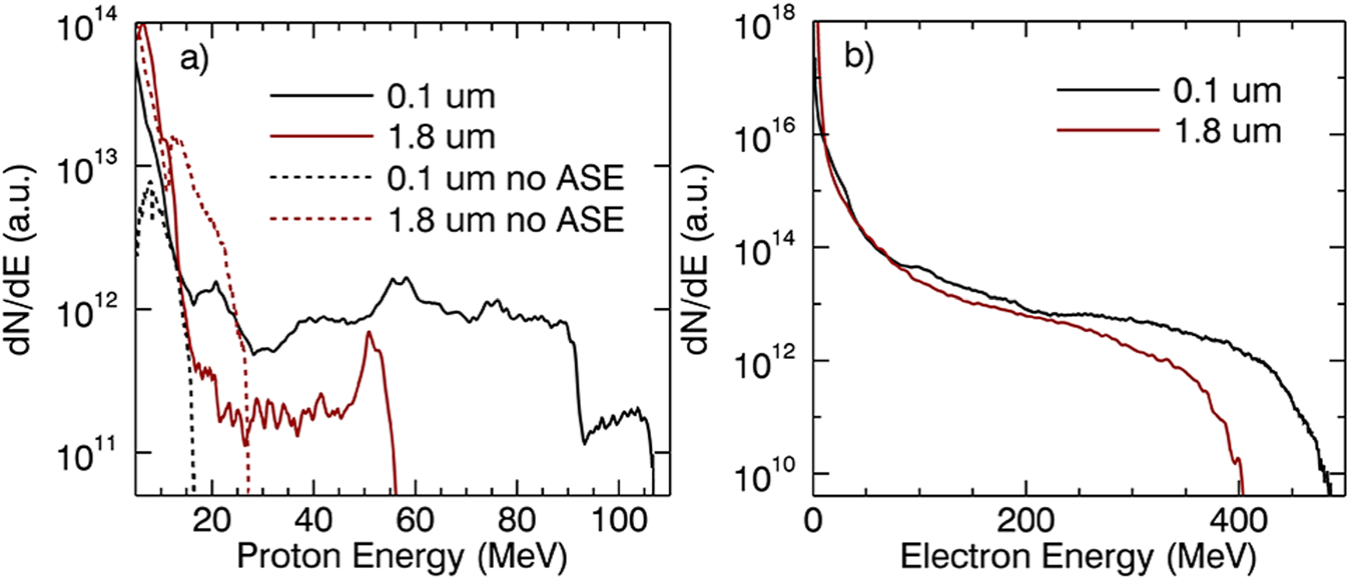
(**a**) Proton energy spectra of 0.1 *μm* and 1.8 *μm* targets with and without ASE at 250 T. (**b**) The electron energy spectra of 0.1 *μm* and 1.8 *μm* targets with ASE at 120 T.

To illustrate the acceleration process of protons in the sheath field, 2D PIC simulations with parameters of plasma density distribution, and electron/proton temperatures obtained from hydrodynamic simulations were performed. In the simulations, the main laser is circularly polarized and has a Gaussian envelope. The laser normalized intensity, spot focus size, and duration were set as 12 (corresponding to a peak laser intensity of 3.08 × 10^20^ *W*/*cm*^2^), 6 *μm* and 10 *T* respectively, where *T* = 2.67 *fs* is the laser period. The transverse and longitudinal electric fields obtained from simulations for 0.1 *μm* and 1.8 *μm* targets for the case of with ASE are depicted in Fig. 3. Relativistic self-focusing and laser intensity enhancement can be observed in both cases as shown in Fig. 3(a). The maximum enhancement factor of laser amplitude is 4.2 for the 1.8 *μm* target, which is slightly larger than that of 4 for the 0.1 *μm* target. In spite of stronger self-focusing, the maximum sheath electrostatic field at the rear side of the 1.8 *μm* target is significantly weaker than that for the 0.1 *μm* target as shown in Fig. 3(b). The proton energy spectra are shown in Fig. 4(a). The maximum proton energy for the 0.1 *μm* and 1.8 *μm* target is about 107 MeV and 56 MeV at 250 T respectively. As an interesting comparison, we also performed simulations for the same targets without ASE. it’s found that the values of maximum proton energy for both targets are significantly lower compared to the cases of with ASE.

## Discussion

The density profile of the target after the interaction of ASE plays a key role in the ion acceleration. For the 0.1 *μm* and 1.8 *μm* targets under the irradiation of nanoseconds ASE, the areal density of the region before the critical density plasma is very small compared to the bulk targets. Thus, we can employ hydrodynamic analytical theories to illustrate the physics and reveal the dependence of density profile on the ASE intensity and duration as below. When the ASE irradiates a target, the plasmas at the target front will expand due to the absorption of laser energy. Here, *l*_*f*_ can be determined by *l*_*f*_ = *c*_*s*_*t*, where *c*_*s*_ is the sound velocity of the expanding plasma. The *c*_*s*_ can be expressed by *c*_*s*_ ≈ 0.31 × 10^6^$\sqrt{\frac{{T}_{e}}{keV}}$ $\sqrt{\frac{Z}{A}}$^45^, where *Z* and *A* are effective ion charge and the mass number. For the ASE intensity of ${10}^{11}\sim {10}^{13}W/c{m}^{2}$, *T*_*e*_(*eV*) = ${(\frac{{I}_{ASE}}{{10}^{4}W/c{m}^{2}}{(\frac{\lambda }{{\mu }m})}^{2})}^{2/3}\,eV$. Meanwhile, a strong shock is launched and travels quickly through the target. The shock has the velocity of *v*_*s*_ = $\sqrt{(\gamma +1)\frac{{P}_{s}}{{\rho }_{0}}}$^25^, where *γ* is the adiabatic constant, *ρ*_0_ is the density of the target, and *P*_*s*_(*Mbar*) = 8.6${(\frac{{I}_{ASE}}{{10}^{14}W/c{m}^{2}})}^{2/3}{\lambda }^{-2/3}{(\frac{A}{2Z})}^{1/3}$ is the shock pressure. If the target is thin enough that the shock can arrive at the target rear and break out within the ASE duration, a rarefaction wave will be created. The target will be ionized in a very short time by the rarefaction wave and form a dense plasma moving forwardly driven by the shock pressure. In an overall point of view, the target is pushed as a whole by the shock pressure of ASE. For simplicity, the mean velocity of the target can be approximately given by *v*_*target*_ ≈ $\frac{1}{2}\frac{{P}_{s}}{{\rho }_{0}d}({\tau }_{ASE}-\frac{d}{{v}_{s}})$^25^, where *d* is the thickness of the target. After the interaction of ASE, the displacement of the target is *x* ≈ $({\tau }_{ASE}-\frac{d}{{v}_{s}})\times {v}_{{target}}$.

Based on the above analysis, the condition that leads to the formation of an plasma density profile with a long extension at the target front and a sharp gradient at the target rear after the ASE would be

1 $$x > {c}_{s}{\tau }_{ASE}.$$

In this case, the whole target will catch up with the plasma and ‘strike’ it. Accordingly, a plasma density profile of the target after the ASE, with a sharp plasma gradient at the target rear, will be formed. By plugging in the expression of *c*_*s*_ and *x*, we can get:

2 $$d\le \frac{{P}_{s}{\tau }_{ASE}}{2{\rho }_{0}{c}_{s}(1+\frac{1}{{v}_{s}})}.$$

Accordingly, there is a critical target thickness of *d*_*c*_ = ${d}_{c}=\frac{{P}_{s}{\tau }_{ASE}}{2{\rho }_{0}{c}_{s}(1+\frac{1}{{v}_{s}})}$ where the regime just takes place. Take ASE intensity of 10^12^ *W*/*cm*^2^, *τ*_*ASE*_ = 0.5 *ns* and *λ* = 800 *nm* as an example, *P*_*s*_ and *c*_*s*_ are about 0.41 *Mbar* and 30 *km*/*s* respectively. For the Al target, we obtain *d* ≈ 0.13 *μm*. The 0.1 *μm* target meets the demand of forming an symmetric plasma density profile. Detailed evolution of the plasma density profiles for the 0.1 *μm* target was investigated by hydrodynamic simulations. Results show that *l*_*d*_ increases in a very short time. Then, the peak density of the target drops quickly and the position of density peak starts to move forward. Meanwhile, a plasma density profile with a long extension at the target front and a sharp gradient at the target rear is formed. *l*_*d*_ begins to decrease until *t* = 0.5 *ns*. For the 1.8 *μm* target, the thickness of the target is much larger than *d*_*c*_, and the displacement of the target is very small compared to *c*_*s*_*τ*_*ASE*_. As a result, in contrast to the case of 0.1 *μm* target, the density profile of 1.8 *μm* target has a large *l*_*d*_ under the irradiation of ASE as shown in Fig. 1(a).

The displacement of the targets under ablation pressure also has important influence on *l*_*f*_. The relationship can be expressed as *l*_*f*_ ≈ *x* + *c*_*s*_*τ*_*ASE*_. In the condition of the same laser parameters, *x* of the 0.1 *μm* and 1.8 *μm* targets are calculated as 20 *μm* and 0.3*μm* respectively. This explains why *l*_*f*_ of 0.1 *μm* target with the value of 62 *μm* is slightly large than that of the 1.8 *μm* target, in spite of similar expansion velocity of the preplasma at the target front. The validity of 1D hydrodynamic simulation results is testified by MULTI 2D by using a Gaussian laser. We found that plasma scale length at the rear side of 0.1 *μm* target in the center of the Gaussian laser beam is also significantly smaller than that of 1.8*μm* target in spite of different deformation due to the laser intensity distribution.

The preplasma resulting from the ASE has huge influence on the proton acceleration. When interacting with the preplasma at the target front, the main laser pulse experiences relativistic self-focusing due to the spatiotemporal variation of the refractive index and a long-living channel is formed as shown in Fig. 3(a). The radius of the focused laser spot size can be estimated as *r* = $\frac{1}{\pi }\sqrt{\frac{{a}_{0}{n}_{c}}{\sqrt{2}ne}}$^28,29,33–35,46^, where *n*_*e*_ is the initial electron density. The *n*_*e*_ of the 0.1 *μm* and 1.8 *μm* targets is about 0.37 *n*_*c*_ and 0.4 *n*_*c*_ respectively, calculated as the mean density of the preplasma by the method of area integral. For our simulation parameters, this gives the laser spot size value of 1.55 *μm* and 1.5 *μm*, consistent with the simulation results (1.52 *μm* and 1.45 *μm*). In addition, the preplasma length of the two targets approach to the self-focusing length with the value of 55 *μm* shown in 3(a). This ensures an efficient self-focusing. When the self-focusing happens, the laser amplitude is enhanced, such as in our cases, by a factor of 4 and 4.2 for the two types of targets. At the same time, the energetic electrons are efficiently accelerated by the direct laser acceleration (DLA)^47–49^. The temperature of the DLA electrons can be described as *T*_*e*_ = 1.5(*I*_*main*_*λ*^2^/13.8*GW*)^1/2^, where *I*_*main*_ is the intensity of the main pulse. The maximum energy of the DLA electrons is more than 400 MeV as shown in Fig. 4(b). This is obviously larger than the electrons accelerated by the ponderomotive potential described as *T*_*e*_ = 0.5[(1 + (*I*_*main*_*λ*^2^/13.8*GW*)/2)^1/2^ − 1]^28,29,33,46^. These DLA electrons have high energy density. When they arrive at the target rear, a stronger and stable sheath field is bulit, resulting in a higher proton energy compared to the case of without ASE.

**Figure 5:**
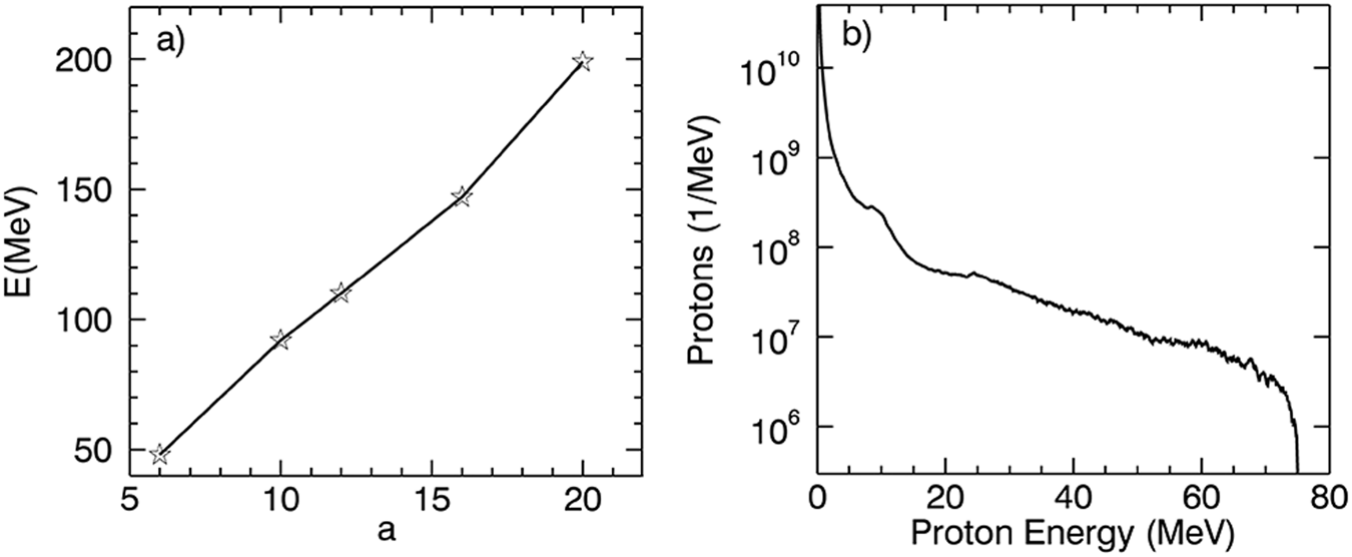
(**a**) The scaling law between the proton energy and the laser intensity. (**b**) Proton energy spectra of a 0.1*μm* target obtained from 3D simulations.

We note that the maximum proton energy of 0.1 *μm* target is about twice as much as that of 1.8 *μm* target with presence of ASE. The two targets have the similar preplasma profile at the target front, resulting in almost the same relativistic self-focusing and laser intensity enhancement. However, plasma scale length at the rear side of 1.8 *μm* target is significantly larger than 0.1 *μm* target. To explain this result, the proton acceleration mechanism of the 0.1 *μm* target needs to be considered. In Fig. 4(a), the protons show a plateau energy spectrum^10,33,50^, which can be judged as TNSA mechanism in previous works. To verify the scheme, a serial of 2D simulations with different intensity of the main laser pulses were performed. Figure 5(a) shows the dependence of maximum proton energy on the laser amplitude. The scaling can be approximately described as *E* ∝ *I*^1/2^, which also shows a remarkable signature of TNSA mechanism in another aspect. For TNSA mechanism of proton acceleration, the maximum proton energy is determined by the sheath electrostatic field at the target rear. The strength of the field can be described as *k*_*B*_*T*_*e*_/(*eλ*_*D*_), where *k*_*B*_, *e*, and *λ*_*D*_ are Boltzmann constant, charge of the electrons, and the Debye length respectively. If the plasma pre-expansion is negligible, the sheath field will be reduced to *k*_*B*_*T*_*e*_/(*el*_*d*_)^17^. Since *l*_*d*_ of the 0.1 *μm* target is significantly smaller than that of the 1.8 *μm* target, the maximum proton energy obtained from the 0.1 *μm* targets is consequently higher.

For a more precise prediction of proton energy, 3D PIC simulations with the same plasma density and laser parameters were performed. The proton energy spectra is shown in Fig. 5(b). The maximum energy of the protons is 70 MeV, lower than the results in 2D simulations.The difference of the maximum proton energy between the 2D and 3D simulations is due to the 3D effect. For example, the electrons beam expansion spatial angle is 2 *π* in 2D but 4 *π* in 3D space; the sheath field in 3D simulations will be lower owing to the faster expansion of the electrons. All these are overlooked in 2D simulations.

In conclusion, we have demonstrated the enhanced proton acceleration from hundreds nanometer target irradiated by a normal contrast (10^7^–10^9^) laser pulse containing nanosecond plateau ASE. It is shown in 2*D* simulations that protons with the energy more than 100 MeV can be generated by a Gaussian CP laser pulse at the intensity of 3.08 × 10^20^ *W*/*cm*^2^ interacting with a 0.1 *μm* aluminum target. Compared to the case using 1.8 *μm* targets, the maximum proton energy is 2 times higher. We reveal that such enhancement is due to the plasma profile with a long extension at the target front and a sharp gradient at the target rear created by the plateau ASE. It provides an efficient way to obtain high energy protons for the wide applications in the condition of laser containing nanosecond plateau ASE, instead of employing complicated methods to increase the contrast. We should emphasize that the scheme is verified for the main pulses with plateau ASE. The extendibility of our conclusions to other types of ASE with prepulses or exponential rising edge needs to be further studied.

## Methods

### Hydrodynamic Simulations

Hydrodynamic code MULTI was employed to obtain the evolution and the density distribution of the targets irradiated by the ASE. The ASE pulse has the duration *τ*_*ASE*_ = 0.5 *ns*, intensity ranging from 10^11^ *W*/*cm*^2^ to 10^13^ *W*/*cm*^2^ and wavelength *λ* = 0.8 *μm*. The material of the targets is aluminum and its initial density *ρ*_0_ is 2.7 *g*/*cm*^3^.

### PIC Simulations

PIC simulations of the proton acceleration for Al targets with the thickness of 0.1 *μm* and 1.8 *μm* were performed at the cases of presence and absence of ASE. The fully relativistic 2D PIC code, EPOCH, was used. Each simulation was defined with Cartesian spatial dimensions of 100 *μm* × 30 *μm* using 12000 × 600 computational mesh cells. A circularly polarized laser pulse with a Gaussian envelope $a={a}_{0}\exp (-\frac{{(x-{x}_{0})}^{2}}{{r}_{0}^{2}})\exp (-\frac{{(t-{t}_{0})}^{2}}{{\tau }^{2}})$ irradiates the target normally from the left side. The laser parameters *a*_0_,*x*_0_,*r*_0_,*t*_0_, and *τ* are set as 12 (corresponding to a peak laser intensity of 3.08 × 10^20^ *W*/*cm*^2^), 0,6 *μm*,20 *T* and 10 *T* respectively, where *T* = 2.67 *fs* is the laser period. The targets in simulations were Aluminum materials with the initial density of 2.7 *g*/*cm*^3^. We set the target two ion species (aluminum ions and protons) with the ratio of 10:1. Simulations of the ion acceleration for the target with the thickness of 0.1 um and 1.8 um are performed at the cases of low-contrast and high-contrast laser (with and without ASE pedestal). The boundaries of the simulation box are all defined as free space. The laser enters from the left boundary. 3D PIC simulations with box size of 100 *μm* × 30 *μm* × 30 *μm* sampled by 5000 × 450 × 450 grids were performed.
